# Impulsive Choice Predicts Poor Working Memory in Male Rats

**DOI:** 10.1371/journal.pone.0093263

**Published:** 2014-04-14

**Authors:** C. Renee Renda, Jeffrey S. Stein, Gregory J. Madden

**Affiliations:** Department of Psychology, Utah State University, Logan, Utah, United States of America; Université Lyon, France

## Abstract

A number of maladaptive behaviors and poor health outcomes (e.g., substance abuse, obesity) correlate with impulsive choice, which describes the tendency to prefer smaller, immediate rewards in lieu of larger, delayed rewards. Working memory deficits are often reported in those diagnosed with the same maladaptive behaviors. Human studies suggest that impulsive choice is associated with working memory ability but, to date, only one study has explored the association between working memory and impulsive choice in rats and no relation was reported. The current study reevaluated the association between working memory and impulsive choice in 19 male Long-Evans rats. Psychophysical adjusting procedures were used to quantify working memory (titrating-delay match-to-position procedure) and impulsive choice (adjusting delay procedure). Rats were partitioned into low- and high-impulsive groups based on performance in the impulsive choice task. Low-impulsive rats performed significantly better in the working memory assessment. Across all rats, impulsive choice was negatively correlated with working memory performance. These findings support the hypothesis that prefrontal cortex function, specifically, working memory, is related to impulsive choice. Future research might profitably examine the experimental variables designed to influence working memory to evaluate the effects of these variables on impulsive choice and maladaptive behaviors with which it is correlated.

## Introduction

Impulsivity is a multi-dimensional construct describing behavioral tendencies to act prematurely, engage in risk-taking and sensation-seeking, to make impulsive choices, etc. [1]. Impulsive choice describes an organism's preference for a smaller-sooner reward (SSR) over a larger-later reward (LLR). Delay discounting is the behavioral process thought to underlie impulsive choice (i.e., the devaluation of a reward as the delay to its receipt increases). A good deal of interest in delay discounting derives from the robust, positive correlation between steep discounting and substance dependence/abuse [2] and emerging evidence that steep delay discounting is correlated with obesity [3], pathological gambling [4], [5], risky drug use [6], and risky sexual behavior [7]. The nature of these correlations is not well understood, with evidence suggesting that steep delay discounting both precedes and predicts drug use [8]–[10], and that chronic drug use yields neuroadaptations that increase delay discounting [11]. Likewise, an unexplored third variable may account for the correlation between steep delay discounting and drug use [12], [13].

The finding from fMRI studies that increased prefrontal cortex activity is associated with lower levels of impulsive choice [14] has led researchers to explore the relation between prefrontal cortex deficits, maladaptive behavior, and delay discounting [15], [16]. Of course, the number of behaviors that are mediated by the prefrontal cortex and may be relevant to impulsive choice is large (e.g., attention, response inhibition, future planning, self-monitoring, working memory [17]–[20]). For six reasons, the present paper focuses on the relation between working memory and delay discounting. First, some of the maladaptive behaviors associated with steep delay discounting are also associated with poor working memory. These include drug abuse [21]–[23], obesity [24] and pathological gambling [25]. Second, among humans, poor working memory ability is correlated with steeply discounting delayed rewards [9], [26], [27]. Third, using activation likelihood estimation, Wesley and Bickel [28] pooled data from neuroimaging studies of delay discounting and working memory. Overlap analyses between working memory and delay discounting, independent of shared activity between two control conditions (response inhibition and finger tapping), revealed large activity clusters in the left lateral prefrontal cortex that were unique to working memory and delay discounting. Fourth, taxing working memory may increase impulsive choice [29] (although see [30] for an alternative account of this finding). Fifth, evidence suggests that transcranial magnetic stimulation designed to disrupt the functioning of the dorsolateral prefrontal cortex impairs working memory [31] and increases impulsive choice [32], although the latter studies are not without exception [33], [34]. Six, one study has demonstrated that improving working memory reduces impulsive choice among stimulant-dependent individuals [26]. This finding is consistent with the hypothesis that working memory deficits can underlie preference for SSR over LLR [35] and, more broadly, that executive-function deficits underlie steep delay discounting and addiction (see the *competing neurobehavioral systems hypothesis of addiction* [16]).

The present study was designed to further evaluate the relation between working memory and delay discounting, the former defined as “the active maintenance and flexible updating of goal/task relevant information…in a form that has limited capacity and resists interference” [36]. The primary reason for studying this relation in rats was to evaluate the cross-species generality of the relation observed in humans. Establishing this relation may open important lines of research (e.g., evaluating the effects of working-memory training on delay discounting and addiction-related behavior). To date, only one study has examined the relation between working memory ability and delay discounting in rats [37]. Dellu-Hagedorn [37] reported that high-impulsive (HiI; n = 10) and low-impulsive rats (LoI; n = 10) did not differ in the number of errors made in an eight-arm radial maze (a widely used test of rodent working memory).

The present study employed different procedures for assessing working memory and delay discounting. A titrating-delay match-to-position task was used to evaluate working memory; the task required active maintenance of task-relevant information (the location of the sample stimulus) while completing an interference task (rear-lever responding) during the retention interval. To maximize individual differences in both the working-memory and delay-discounting tasks, we selected psychophysical titrating procedures that placed no upper bound on the duration of the retention interval (working memory) or the delay to the LLR (discounting task).

## Method

### Ethics Statement

This study was conducted in accordance with the procedures and policies of the Animal Welfare Act. Approval was granted by the Institutional Animal Care and Use Committee at Utah State University (protocol number: 2232).

### Subjects

Subjects were 19 experimentally naïve, male Long-Evans rats (Harlan, Indianapolis, IN). The rats were approximately 75 days old at intake and were housed individually in polycarbonate cages within a colony room operating on a 12-hr light/dark cycle (light onset at 7:00 am). Water was freely available in the home cage but food intake was restricted to maintain the rats at 85% of the dealer supplied growth curve free-feeding weight.

### Apparatus

Sessions were conducted in 19 identical operant chambers (Med-Associates, St. Albans, VT), each equipped with a white-noise speaker and housed within a sound-attenuating cube. A food receptacle was centrally positioned on the front wall of the chamber (6 cm above the grid floor), into which a pellet dispenser, mounted outside the chamber, delivered 45-mg food pellets (Bio-Serv, Frenchtown, NJ). One of two retractable, low-profile levers was positioned on either side of the receptacle (10.5 cm above the grid floor). An identical lever was located centrally on the rear wall (10.5 cm above the grid floor). A 28-V DC cue light was positioned above each lever.

### Procedures

**Figure 1:**
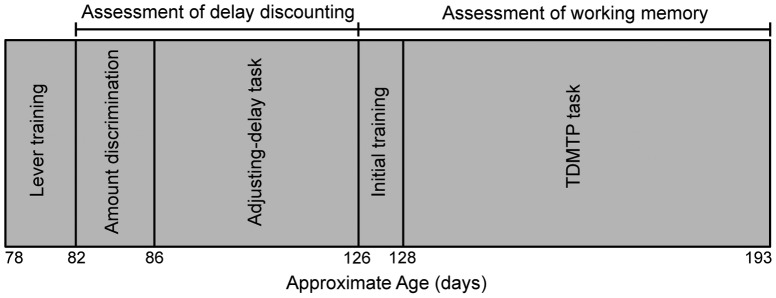
Schematic representation of experimental conditions across age. Age varied slightly across sessions due to mastery-based criteria in training.

Figure 1 depicts the sequence and approximate duration of the experimental conditions.

#### Lever training and amount discrimination

An autoshaping procedure was used to establish lever pressing. Once reliable lever pressing was observed, subsequent training sessions were conducted to provide rats with exposure to the chained schedules of reinforcement used in subsequent tasks. These lever-training sessions were composed of 80 trials, each separated by a fixed, 20-s intertrial interval (ITI). Initially, trials began with the insertion of either the left or right lever and illumination of its associated cue light (left-right order alternated strictly across trials). During these sessions and in all procedural tasks described below (unless otherwise noted), a single lever press caused the lever to retract, its cue light to darken, and the next event in the trial sequence to be initiated. Following a press on the active lever, two food pellets were immediately delivered to the receptacle. After completing ≥90% of the trials for two consecutive sessions, the insertion of the rear-wall lever initiated the trial. In this case, following a press on the rear-wall lever, one of the side levers on the front wall was inserted and its cue light was illuminated. Failure to respond within 10 s on any lever was counted as an omission and initiated the ITI. Lever training ended when the rat completed ≥90% of the arranged trials for two consecutive sessions.

Following lever training, an amount-discrimination task was used to ensure that the rats could discriminate reward amount in the absence of delay. Sessions were composed of 60 trials, partitioned into 15 blocks of 4 trials each. Each trial block began with two forced-choice trials in which, following a single press on the rear-wall lever, only one lever (left or right) and its associated cue light was presented on the front wall (order randomly determined without replacement). A single press retracted the lever, turned off the cue light, and delivered either a 1- or a 3-pellet reward, depending on the lever that was pressed; lever assignment was counterbalanced across subjects. The final two trials of each trial block were free-choice trials in which both front-wall levers (and cue lights) were presented after a response to the rear-wall lever. The reward amount delivered for a single lever press was the same as in the forced-choice trials. Following food delivery, a variable-duration ITI was activated that ended 90 s after the preceding trial began. On all trials, a limited-hold contingency was imposed, such that if a lever press did not occur within 20 s of its insertion, that trial was counted as an omission. To ensure consistent exposure to the contingencies on both levers, omitted forced-choice trials were repeated after the ITI elapsed; omitted free-choice trials were not repeated. This task ended when the rat selected the 3-pellet reward on ≥90% of the trials for two consecutive sessions.

#### Assessment of delay discounting

To quantify delay discounting, an adjusting-delay procedure was used [38]. With the following exceptions, procedures were identical to those programmed in the amount-discrimination task: A delay (initially 0 s) was imposed between pressing the lever associated with the 3-pellet reward and delivery of the pellets. The duration of the delay to the LLR was adjusted between trial blocks, depending on free-choices made within the preceding trial block. If the LLR was selected on both free-choice trials, the delay was increased by 1 s in the next trial block; selecting the SSR on both free-choice trials decreased the delay by 1 s. Selecting each option within a trial block resulted in no change to the delay. The delay programmed at the beginning of a session was that which was in effect at the end of the preceding session. During the delay to the LLR, the cue light above the selected lever remained illuminated. A programming error illuminated the lights above both levers for the first 19–25 sessions. An additional 20 sessions were conducted after this error was fixed.

#### Assessment of working memory

Following the adjusting-delay task, rats were exposed to sessions featuring the basic trial structure and response requirements used in the working-memory task. Trials began with the random insertion of either the left or right lever on the front wall and illumination of its associated cue light. Over the course of several sessions, the response requirement on this lever was increased from 1 to 10 presses. In each session, once the response requirement had been met, the lever on the rear wall was inserted and its associated cue light was illuminated. Pressing this rear-wall lever once delivered two food pellets to the receptacle. Trials were separated by a fixed, 20-s ITI in which the white-noise speaker was activated. To signal the upcoming trial, the white-noise speaker cycled on and off at 0.25-s intervals during the final 3 s of the ITI.

Next, a modified titrating-delay match-to-position (TDMTP) procedure was used to quantify the working-memory capacity of each rat. Although delayed matching-to-position procedures are frequently used in rodent studies of working memory [39], we modified the task so that rats were required to press the rear-lever during the retention interval [40]. This rendered the task closer to the NIMH [36] definition of working memory, as rats had to actively maintain task relevant information while completing the rear-lever task that may interfere with this maintenance. The rear-lever task also served to disrupt any mediating behavior that might have otherwise occurred during the retention interval (e.g., holding a position near the sample lever); observations made in a sample of TDMTP sessions revealed no evidence of mediating behavior.

Trials began with the insertion of a randomly selected lever on the front wall (i.e., the “sample lever”) and illumination of its cue light. Each sample lever was presented an equal number of times in the session and the same sample could not be presented more than four times consecutively. Following ten presses on the sample lever [41], the rear-wall lever was inserted, its cue light was illuminated, and the retention-interval timer was initiated. A single response on the rear lever after the retention interval elapsed presented both levers (and their associated cue lights) on the front wall (i.e., the “comparison levers”). Pressing the comparison lever that had previously been presented as the sample was counted as a correct response and resulted in delivery of two food pellets to the receptacle; pressing the opposite lever was counted as an incorrect response and did not result in pellet delivery. A fixed, 20-s ITI, with white noise accompaniment, followed correct and incorrect responses. As in initial training, the white-noise speaker was turned on and off every 0.25-s during the final 3 s of the ITI.

Limited-hold contingencies of 25 s (sample-lever response requirement) and 10 s (rear- and comparison-lever presses) were imposed throughout this phase. If the limited hold elapsed before the lever was pressed, the 20-s ITI was initiated. A correction procedure was employed throughout, such that omitted trials or trials in which an incorrect comparison lever was selected were repeated until the correct lever was pressed [42].

The titrated retention-interval duration served as the measure of working-memory capacity of each rat. The first time that comparison-lever selection was ≥90% correct over the preceding 20 trials, the retention interval was increased from 0 to 0.25 s. Subsequently, percentage correct was calculated over a moving window of the preceding 20 trials (excluding correction trials and reading into the preceding session if necessary). Retention-interval titration opportunities occurred every eighth trial. If the percentage correct was ≥90%, the retention interval was increased by 0.25 s or 2% whichever was largest. If the percent correct was <70% overall, or <70% on either lever, the retention interval was decreased by 0.25 s or 2%; otherwise, no change was made.

The TDMTP task was conducted for 65 sessions, each composed of 48 trials (excluding correction trials). During eight of these trials, the retention interval was 0 s; these 0-s trials have been shown to decrease sample omissions and improve accuracy [43], [44] and they encourage rear-lever responding early in the retention interval (thereby decreasing the probability of mediating behavior). These 0-s retention-interval trials occurred pseudorandomly with the constraint that no more than two of these trials could occur consecutively.

#### Data analysis

Mean adjusted delay (MAD) for each rat calculated over the final nine sessions of the adjusting-delay task served as our measure of delay discounting, wherein MAD is inversely related to the degree of delay discounting. To compare differences in working memory across extreme groups, rats were partitioned into two groups composed of the 7 least impulsive (LoI, n = 7) and the 7 most impulsive (HiI, n = 7) rats. Independent t-tests were used to evaluate between group differences with alpha set to .05. Working memory ability was quantified as the final retention interval obtained in each session, with longer retention intervals reflecting better working memory. Bartlett's Test of Sphericity was used to examine intercorrelations of working memory performance across sessions. For the correlations, terminal MAD values and retention intervals were natural log-transformed to meet the assumptions of parametric statistics. A constant of 0.01 was added to all numbers prior to data transformation to avoid division by zero. Pearson correlations were used to assess the relationship between retention intervals and terminal MADs. To control the familywise error rate, a Bonferroni correction was applied to the three correlations evaluated for significance; the correction used a criterion alpha of .02.

## Results

**Figure 2:**
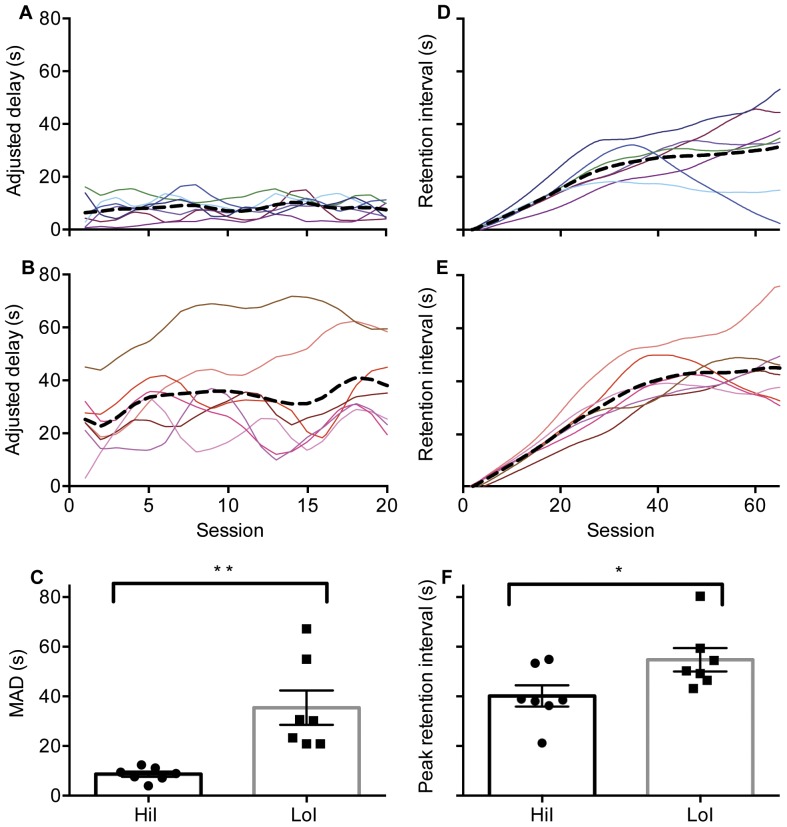
Adjusted delays and retention intervals. *Panels A–B.* The solid data paths represent average adjusted delays over the final 20 sessions of the adjusting-delay task for HiI and LoI rats, respectively. *Panel C.* Terminal mean adjusted delays (MADs) for HiI and LoI rats. *Panels D–E.* Each colored data path denotes a different subject's changing retention interval over the course of the TDMTP task for HiI rats and LoI rats, respectively. The dashed black data paths show the mean retention interval for each group. *Panel F.* Average peak retention intervals for HiI and LoI rats. * indicates significance at p<.05; ** indicates significance at p<.01. Error bars depict *SEM*.

Figures 2A (HiI rats) and 2B (LoI rats) show spline fits to individual adjusted delays over the final 20 sessions of the adjusting delay procedure. The black dashed data paths show the average adjusted delay for each group. As illustrated in Figure 2C, terminal MADs were significantly longer in the LoI than in the HiI group, *t* = 3.83, *p*<.01. No significant differences were observed across groups in forced- or free-choice omissions or latency to make a response across the final 20 sessions, *p*>.10 in all cases (data not shown).

Figure 2D and 2E show spline fits to HiI and LoI rat's, respectively, titrating retention intervals over the 65 sessions of the TDMTP procedure. Recall that retention intervals increased with accuracy, so retention intervals serve as a metric of working memory ability. Across both groups of rats, a significant linear increase in retention intervals reveals that working-memory performance tended to improve over the 65 sessions, *p*<.0001. Inspection of the individual rats' data reveals several divergent patterns common to both groups. For five rats (3 HiI and 2 LoI), retention intervals tended to increase over the 65 sessions of the TDMTP task. For another six rats (3 HiI and 3 LoI), a retention interval peak was reached, after which retention intervals stabilized. For three rats (1 HiI and 2 LoI), a retention-interval peak was reached after which they declined precipitously as delayed match-to-position accuracy declined. For two of the rats displaying this latter pattern (1 HiI and 1 LoI), the decline occurred because the rats demonstrated a bias toward selecting one of the comparison-stimulus levers regardless of the sample stimulus location; the decline in the other rat was unrelated to bias. Figure 2F depicts the average peak retention interval obtained for both groups. There was a significant difference in retention interval between groups, *t* = 2.29, *p*<.05. No significant differences were observed in omissions, number of trials completed, or latency to respond across the final 20 sessions, *p*>.10 in all cases (data not shown).

**Figure 3:**
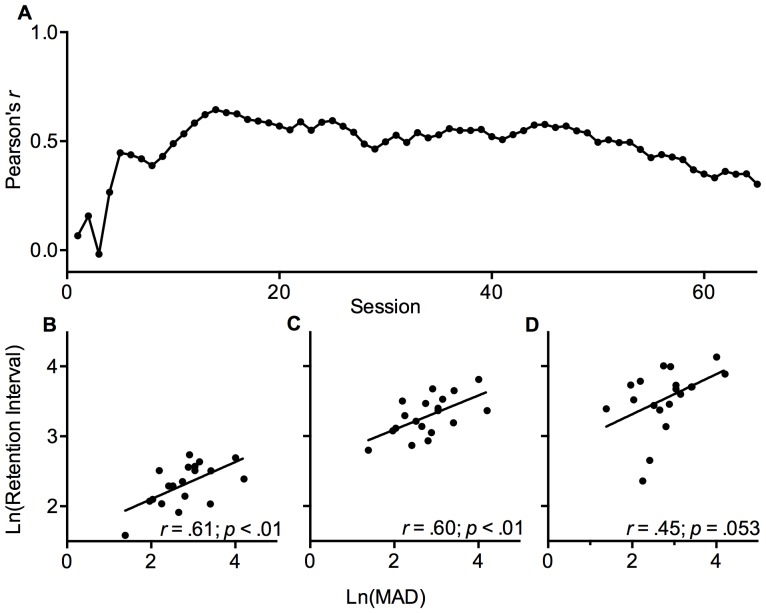
Correlation between mean adjusted delay (MAD) and retention intervals. *Panel A.* Pearson *r* correlation coefficients between ln-transformed terminal MADs and ln-transformed retention interval at the end of each session of the TDMTP task. *Panels B–D.* Scatterplots depicting the relation between ln-transformed terminal MADs and the average ln-transformed retention intervals observed across sessions 10–16 (Panel B), sessions 17–45 (Panel C), and sessions 46–65 (Panel D). Pearson's *r* coefficients are provided in each panel. The criterion alpha value after Bonferroni correction was .02.

Figure 3A explores the relation between all rats' terminal MAD values in the delay-discounting task and session-by-session retention intervals in the TDMTP task. In this exploratory analysis, Pearson's *r* coefficients increased as individual differences in working-memory ability emerged (i.e., as LoI rats performed better on the test of working memory) and retention intervals were increasingly correlated with terminal MAD values. Figure 3B shows a significant correlation between MAD values and retention intervals (*r* = .61, *p*<.01) as the latter were increasing but had not yet reached peak levels (sessions 10–16). Across sessions 17–45 the slope of a linear function fit to the *r* coefficients did not significantly deviate from zero (Figure 3A) and retention intervals across this range were highly intercorrelated, *X* ^2^ = 372.68, *p*<.001. As shown in Figure 3C, the correlation was significant between terminal MAD values and mean retention intervals across this range of sessions in which peak retention intervals tended to be reached, *r* = .60, *p*<.01. From sessions 46–65, *r* coefficients declined slightly due, in part, to some rats' deteriorating working memory performance. Because measures of motivation (response latencies and trial omissions) were unchanged across this final range of sessions (*p*>.05), we examined the correlation between MAD values and mean retention intervals across this final range the correlation approached significance, *r* = .45, *p* = .053 (Figure 3D).

## Discussion

In the present study, the relation between delay discounting and working memory performance in rats was examined. On average, HiI rats exhibited poorer working memory performance as indicated by significantly lower retention intervals compared to rats in the LoI group. Across all rats, longer MAD values, reflective of fewer impulsive choices made in the delay-discounting task, were predictive of better performance, and hence, longer retention intervals in the test of working memory.

This finding contrasts with the lack of a correlation between working memory and impulsive choice in rats reported by Dellu-Hagedorn [37]. Procedural factors may help explain why our findings are different than those reported by Dellu-Hagedorn. To assess working memory, Dellu-Hagedorn used an eight-arm radial maze that required rats to retain multiple pieces of information (i.e., previously visited arms) in working memory, whereas the TDMTP procedure only required retention of one stimulus per trial. In addition, our procedures placed no upper limit on the dependent measures used to quantify impulsive choice (MAD under the adjusting-delay task) or working memory (retention interval under the TDMTP task), whereas these measures were bounded in the Dellu-Hagedorn study. The current findings are consistent with the human literature [14], [26], [27], [29], [32] and with the competing neurobehavioral systems hypothesis of addiction [16].

In one of the more interesting studies evaluating the relation between working memory and delay discounting, Bickel et al. [26] arranged for a group of stimulant-dependent individuals to complete either working-memory or sham-control training. In comparing pre- and post-training measures of delay discounting, those who received working memory training discounted less steeply the value of delayed monetary outcomes; the sham-control group, by contrast, demonstrated no significant pre- to post-training shift in discounting. This finding is consistent with the direction of the correlation reported in the present study—better working memory skills were predictive of less discounting of delayed rewards. One shortcoming of the Bickel et al. study is that post-tests of working memory did not improve following working-memory training, an outcome the authors attributed to insensitive post-test measures. As illustrated in Figures 2D and 2E of the current paper, our rats demonstrated substantial improvements in working memory over the 65 days in which they completed the TDMTP task. Indeed, one factor in the decreasing correlation between MADs and working-memory performance at the end of the study was that rats the initially performed poorly in the test of working-memory improved their ability with continued exposure to the task. If, in a future study, the present methods were accompanied by an appropriate control group and a post-test of delay discounting one could evaluate the inter-species generality of the effect of working-memory training on delay discounting that was reported by Bickel and colleagues.

The hypothesized relation between working memory and delay discounting has been largely driven by the observations that a) activation of the human prefrontal cortex is correlated with diminished impulsivity in delay-discounting tasks [14] and b) steep delay discounting is correlated with lower general intelligence [27]. Shamosh et al. [27] reported that working-memory related activation of the human left aPFC was negatively correlated with delay discounting (i.e., those making more impulsive choices tended to have lower activation of the left aPFC). As noted by Shamosh et al., the left aPFC is involved in information integration rather than information retention or manipulation, all of which are commonly taxed in tests of working memory. Integrating information about reward amount and delay to arrive at a discounted value of the reward is assumed in the structure of a variety of delay discounting equations that have been proposed (e.g., the hyperbolic discounting equation [38]). Further, relational information-integration to assess the relative value of the two reward options is required in delay discounting tasks, regardless of the species employed.

Killeen [35] suggested a less complex role for memory in nonhuman discounting of delayed rewards, and it should be considered here as an alternative mechanism underlying the correlation reported in the present study. Specifically, Killeen suggested that when the LLR was delivered, a failure to remember which response initiated the delay to the LLR (i.e., pressing the lever associated with the LLR) would discourage the formation of an association between the choice response and the delayed reward. If the response-LLR association were poorly established, relative to selecting the SSR (wherein the reward is proximal to the response), then impulsive choices would dominate. Because poorer performance on the TDMTP task was correlated with having previously made more impulsive choices, our findings are consistent with Killeen's account of nonhuman delay discounting—rats with poorer working memory abilities may have more poorly acquired the response-LLR association. As such, the relation between working memory and delay discounting that we observed in nonhumans when real delays to real rewards were arranged may be mediated by a different behavioral process than is the correlation between working memory and delay discounting in human studies that arrange verbal descriptions of prospective rewards.

One limitation of the current study is its small sample size. Failure to detect a significant relationship between delay discounting and the terminal working memory performance might be attributed to inadequate power. With the Bonferroni alpha correction, this correlation only approached significance, *p* = .053. Future research should use the present data to inform a power analysis that could not be conducted in advance of the present research given our novel combination of delay-discounting and working-memory procedures. A second limitation is that not all rats' MAD values were stable by the end of the adjusting-delay task. Although MADs assessed in our lab rarely change after 20 sessions, future studies may wish to ensure that each rat's MAD is stable before further assessments are conducted. A third limitation is that four rats reached a maximum retention interval after which a steep decrease was observed. Two rats developed a persistent response bias, favoring one comparison-stimulus lever over the other regardless of the sample stimulus presented at the beginning of the trial. This finding is consistent with others that have reported side biases at long retention intervals [42], [45]. For the other rat, accuracy fell below criterion levels, independent of a side bias, resulting in a reduction in the retention intervals. So that these problems may be more rapidly ameliorated, future studies should explore more sensitive correction contingencies.

Despite the occasional reduction in retention intervals, by session 65 of the working-memory task, all rats' accurate choices led to substantial increases in the retention interval relative to what was observed in the first 10 sessions of training. Indeed, the terminal retention intervals in our study are substantially longer than those produced by comparable procedures in animals given fewer sessions [41], [46], [47]. Thus, the TDMTP procedure used here might be a good choice for future studies evaluating the effects of working-memory training on subsequent delay discounting in rats. As noted previously, such a study would need to include a sham-control group comparable to that employed by Bickel et al. [26].

Whereas the present study is the first to our knowledge to document a relation between working memory and impulsive choice in nonhumans, recent findings from the animal laboratory suggest that working memory is similarly related to another form of impulsivity—impulsive action, or behavioral disinhibition [48]. That working memory appears related to discrete forms of impulsivity reveals a potentially fertile area for future research. Specifically, researchers may wish to use multivariate methods to compare the variance accounted for in working memory performance by each measure of impulsivity.

In conclusion, the current study is the first to demonstrate a correlation between poor working memory and steep discounting of delayed food rewards in nonhumans. Perhaps more importantly, the observed correlations show consistency with previous studies employing human participants. The preparations used herein provide an avenue for examining the generality of other findings reported in the human literature (e.g., working-memory training) and provide the opportunity to examine variables not amenable to human research (e.g., drug self-administration, neurological measures/manipulations).
